# A Community-Based Model to the COVID-19 Humanitarian Crisis

**DOI:** 10.3389/fcimb.2021.639579

**Published:** 2021-03-15

**Authors:** Mirco Nacoti, Andrea Ciocca, Pietro Brambillasca, Francesco Fazzi, Michele Pisano, Massimo Giupponi, Antonio Pesenti, Oliviero Valoti, Maurizio Cereda

**Affiliations:** ^1^Department of Anesthesia and Intensive Care, ASST Papa Giovanni XXIII, Bergamo, Italy; ^2^Coordination, Comunità della Salute, Bergamo, Italy; ^3^1st General Surgery Unit, Department of Emergency, ASST Papa Giovanni XXIII, Bergamo, Italy; ^4^Executive Board, Agenzia di Tutela della Salute, Bergamo, Italy; ^5^Department of Emergency, Anesthesia and Critical Care, Foundation IRCCS Ospedale Maggiore Policlinico, Milan, Italy; ^6^Department of Surgical Pathophysiology and Transplantation, University of Milan, Milan, Italy; ^7^Anesthesiology and Critical Care, Perelman School of Medicine, University of Pennsylvania, Philadelphia, PA, United States

**Keywords:** COVID-19, SARS-CoV-2, Humanitarian crisis, Community, pandemic, PHC, Italy

## Abstract

A multidisciplinary group, mainly from Bergamo region - the epicenter of the COVID-19 pandemic crisis in Italy on march 2020– has developed concept of creating intermediate care facilities and proposes a three-tier model of community-based care, with the goal of reducing hospital admissions, contagion and mortality related to hospital overloading and optimizing human resources.

## Introduction

The fall surge of the COVID-19 pandemic is once again jeopardizing the healthcare systems, the economy, and the social fabric worldwide (Paules et al., 2020). This time, restrictive measures are spelling economic and social disaster (Alwan et al., 2020), while creating herd immunity to SARS-CoV-2 infection by the 2021 vaccination campaign is not nowadays predictable (Anderson et al., 2020a). Western healthcare is hospital-centered but, when the number of admissions soar, facilities are rapidly overwhelmed. In these conditions, substandard care is delivered, other conditions are neglected, and cross-infection of staff and patients become common (Nacoti et al., 2020). However, most COVID-19 patients (about 81%) develop mild-moderate disease and may not require hospitalization (Gandhi et al., 2020; Wu and MCGoogan, 2020). Pandemic crises require simple solutions and a change of perspective toward a concept of community-centered care according to the Alma-Ata definition (World Health Organization, 1978). Facing a deteriorating situation, a multidisciplinary group composed by health policy makers and experts in managing infectious outbreaks and humanitarian crises (mainly from Bergamo, Italy - the epicenter of the COVID-19 pandemic on march 2020) has generated concept of creating intermediate care facilities and proposes a three-tier model of community-based care strategy, with the goal of reducing hospital admissions, contagion, and mortality related to hospital overloading.

## Proposed Strategy to Categorize Patients and Levels of Care

### Grade of COVID-19 Illness

During the terrible Bergamo outbreak, we volunteered home-care to patients who could not be admitted due to lack of hospital beds. This early experience taught us that even patients with mild-moderate hypoxemia - pulse oximetry (SpO2) between 90% and 95% - could be managed outside of the hospital as long as SpO2 monitoring, oxygen therapy, and nutritional support were delivered. Otherwise, and especially if elderly or high-risk, they deteriorated and succumbed due to prolonged hypoxemia, immobility, malnutrition, and dehydration (Faustini and Rutstein, 2020; Walker and Maremont, 2020).

We propose a three-tier approach to manage adult patients with COVID-19 more efficiently and to prevent hospital overload. The strategy leverages a network of general practitioners and home care agencies to manage patients and resources locally while involving nearby communities. It can be adapted to the social and geographic characteristics of the area.

**Figure 1:**
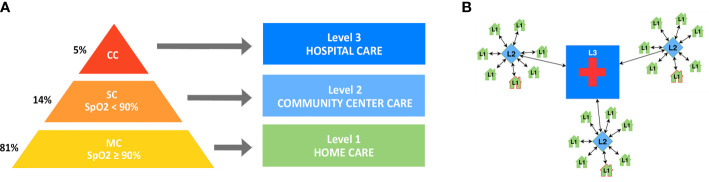
**(A)** Three-tier model of care based on disease severity. **(B)** Overall architecture of the care delivery system. Level 1 (L1) mild or moderate cases (MC) are treated at home; Level 2 (L2) severe cases (SC) receive care in community centers; Level 3 (L3) critical cases (CC) are admitted to the hospital.

Patients are stratified in three grades (Gandhi et al., 2020; Rochwerg et al., 2020) of severity based on resting SpO2 on room air and clinical indicators adapted for limited resources setting from WHO Covid-19 severity categorization (World Health Organization, 2011); each level is linked to specific clinical presentation and treatment responses. A schematic representation is available in the **Figure 1A**:

Grade 1-MILD-MODERATE CASES (MC). Accounting for most cases (81%), this class of patients is defined by SpO2 ≥ 90% and absence of any signs of severe or critical Covid-19; they will receive home care.
Grade 2-SEVERE CASES (SC). This class represents 14% of total cases and is defined by any of SpO2 < 90% on room air, respiratory rate (RR) > 30 breaths per minute, signs of severe respiratory distress (accessory muscle use, inability to complete full sentences). These patients will receive care in intermediate care facilities (Community Centers).
Grade 3-CRITICAL CASES (CC). This class is defined either by lack of response to LEVEL 2 treatment or by the criteria for acute respiratory distress syndrome (ARDS), sepsis, septic shock or other conditions that would normally require the provision of life sustaining therapies such as mechanical ventilation (invasive or non-invasive) or vasopressor therapy; it includes a small fraction of cases (5%) who will receive higher-level or intensive care in hospital units depending on severity and local practice.

Standard protocol for palliative care must be considered at any level according to the geographical and cultural context, age and comorbidities of the patient, occupancy grade of healthcare facilities.

### Level of Care

Patients are stratified in three levels of care (World Health Organization, 2011; Gandhi et al., 2020; Rochwerg et al., 2020), from home care to advanced hospital care, based on the three grades of COVID-19 illness severity. A schematic representation is available in the **Figure 1A**:

*Level 1. Every suspected case with typical symptoms should receive a first visit at home with a dedicated COVID-19 mobile clinic (Yu et al., 2017; International Committee of the Red Cross, 2020) equipped with personal protective equipment (PPE) for clinical, psycho-social and environmental evaluation. We suggest point - of - care rapid antigen testing to identify SARS-CoV-2, allowing early isolation and backward/forward contact tracing. If testing is negative but suspicion for COVID-19 is high, then PCR assay should be performed (Gandhi et al., 2020). A medically sensible approach (Seymour et al., 2020) to these cases suggests:
1. daily monitoring of SpO2, heart rate (HR), temperature and motor activity by telemedicine or with mobile clinics (according to patient preference and local resources) to detect early clinical deterioration in patients with normal SpO2 (≥ 97%);
2. daily monitoring and home care with Oxygen therapy delivered by low flow concentrator, supplemental Nutrition with boosters, resting in the Prone position and Anticoagulation (ONPA) in patients with 90% ≤ SpO2 < 97% and risk factor for severe COVID-19. SpO2 target is 97%.
*Level 2. Every suspected/confirmed case with any of persistent SpO2 < 90% on room air, respiratory rate (RR) > 30 breaths per minute, signs of severe respiratory distress, should be transferred to the COVID-19 community center with a dedicated transport unit to perform a baseline x-ray, electrocardiogram and laboratory evaluation. A medically sensible approach (Faustini and Rutstein, 2020; Gandhi et al., 2020; World Health Organization, 2020a) to these cases suggests: Oxygen therapy (with high-flow concentrator or wall supply), enteral Nutrition if needed, cyclic assisted self-Pronation and Physical therapy, Anticoagulation and Steroids (ONPPAS). SpO2 target is 97%. Personnel and equipment to rescue and transport deteriorating patients should be available. Continuous positive airway pressure (CPAP) or high flow nasal cannula (HFNC) may be considered at this level in high income countries only.
*Level 3. Every suspected/diagnosed case with persistent SpO2 < 90% despite oxygen therapy at the Level 2 settings or with signs of respiratory failure/shock/multiorgan dysfunction at any level should be rapidly transferred to the hospital by a dedicated ambulance. Here, they will receive further inpatient management including more advanced respiratory care.

## Implementation of the Model

The community center is pivotal in this three-tier model (a schematic representation is available in the **Figure 1B**), because it:

*proactively coordinates early detection, isolation (at home or in separate facilities according to baseline environmental evaluation) and tracing at Level 1 (home) care;
*is the place of care for more severe, but not critical cases;
*functions as a triage center for critical cases;
*is crucial when isolation is critical to avoid family cross-contamination;
*may also function as a recovery unit for patients who are early discharged from the hospital;
*may also function as a logistical support for level 1 care providing mobile clinics, oxygen concentrators, drugs, nutrition boosters, personnel protective equipment, cleaning and disinfection facilities.

The proposed strategy should be implemented first in a (pilot) community, led by personnel with experience in the management of epidemics and humanitarian crises (with standardized Infection Prevention Control and logistics strategies) and in collaboration with local health agencies. All involved operators are specifically recruited and trained for the task. In a second phase, the prototype will be replicated on a larger scale and in multiple communities (**Figure 1B**), with qualified personnel dedicated to training, coordination and supervision. Professional staff can be balanced with volunteers (e.g. students) when human resources are strained. The optimal size of each community is comparable to a town or neighborhood (at least 20,000 inhabitants), depending of local characteristics and rate of infection. Public health planning, including social and educational strategy, is required for its sustainable development; the emergency need for assistance can be addressed with *ad hoc* temporary structures, but they must be organized with epidemiological thinking and long-term planning.

## Discussion

Healthcare facilities during the Covid-19 pandemic were stressed (Paules et al., 2020). This situation is forcing a reorganization of the health system with the need to identify simple solutions leading to a concept of community-centered care (World Health Organization, 1978).

With this perspective article, we proposed an innovative three-tier model of community-based strategy of care for Western countries, conceptualizing the creation of intermediate care facilities and inspired by the six core principles of the Primary Health Care (Starfield et al., 2005). The experience of home care during the terrible Bergamo outbreak and new scientific knowledge (World Health Organization, 2011; Gandhi et al., 2020; Rochwerg et al., 2020; Wu and MCGoogan, 2020) permitted us to categorize patients and care in three levels of severity. Aim of this stratification is to avoid hospital overcrowding and identify the most appropriate levels of assistance and locations where therapies can be administered: home care, community centered care, hospital care. Furthermore, the proposed model does not merely outline patient stratification and well-known treatment suggestions, but it also suggests a logistic organization and human resources optimization in case of persistent pandemic. It also underlines the necessity of a team able to educate the workers involved and manage care according to facilities. These non-clinical issues have been identified as key factors involved in suboptimal healthcare in disaster medicine (Sever et al., 2018).

In march 2020, we were heavily involved in the COVID-19 humanitarian crisis in Bergamo, the epicenter of the Italian outbreak; in that period the number of infected people was far higher than what reported by official statistics, which are based on COVID-19 tests performed only in hospitals and on symptomatic patients (World Health Organization, 2020b); in fact, as reported by the local newspaper (Cancelli and Foresti, 2020), the total number of deaths was six times higher than the number recorded in the corresponding segment of 2019. A further study confirmed these narrative reports (Piccininni et al., 2020).When the global medical community face a pandemic of unprecedented scale, with little scientific evidence and “crazy numbers” describing the situation, honest and forthcoming advocacy is an ethical duty. For this reason, a narrative paper was written (Nacoti et al., 2020) aiming a wake-up call for those involved in system preparedness and strategic planning. When evidenced is lacking, narrative medicine is a recognized method of communication (Greenhalgh, 1999) bridging the divide that separate physician and patients, colleagues and society (Charon, 2001). In that narrative manuscript (Nacoti et al., 2020) a change in mindset towards a community-centered approach, that followed the Alma Ata declaration, was advocated (World Health Organization, 1978). The Alma-Ata Conference mobilized a multidisciplinary “Primary Health Care (PHC) movement” that undertook to tackle global health inequalities in all countries with an ambitious program. Forty years later, PHC was reaffirmed as the cornerstone of a sustainable health system for universal health coverage and health-related sustainable development goal (World Health Organization, 2018). Unfortunately, the translation of these values into tangible reforms has been uneven (World Health Organization, 2008) as dramatically evidenced by the present pandemic (Anderson et al., 2020a; Dunlop et al., 2020; Paules et al., 2020). Since its emergence, COVID-19, has become a global health threat (Anderson et al., 2020b). While care in industrialized countries was focused on intensive care and hospital capacity, the COVID-19 pandemic had the characteristics of a global humanitarian crisis (Nacoti et al., 2020).

One year later, the crisis is even more severe. Persistent lockdowns are essential to reduce mortality and buy time to set up pandemic response (Flaxman et al., 2020), but they are disruptive because affect all aspects of human life (Anderson et al., 2020a). Hospital systems in many parts of the world are forced to operate in “crisis capacity” according to the definition of the Institute of Medicine (Hick et al., 2009); oncological programs and even urgent surgery suffering dramatic reduction of the activity (Rausei et al., 2020; Torzilli et al., 2020), diffuse emerging mental health disorders requiring an impressive new effort (Holmes et al., 2020), uncontrolled nosocomial COVID-19 infection in COVID free patients and health care workers (Munster et al., 2020; Nguyen et al., 2020), are some of the key issues stressing daily health systems worldwide. In the meanwhile, creating herd immunity to SARS-COV-2 infection by mass vaccination is very challenging and will require a long and global effort (Alwan et al., 2020). In this context, preparation for resilience of health systems in all countries is still needed and creating intermediate care facilities could be crucial to restore at least a contingency capacity (Hick et al., 2009) of the hospitals, even in cities and towns willing to innovate their care and their pandemic control system. There is clear scientific consensus (John Snow Memorandum) on the Covid-19 pandemic; evidences coming from Japan, Vietnam, New Zealand, Australia show as controlling community spread of COVID-19 is the best way to protect our societies and economies until safe and effective vaccines and therapeutics are available (Anderson et al., 2020a). Furthermore, protecting our economies is inextricably tied to controlling COVID-19. Fort this reason we must protect our workforce and avoid long-term uncertainty.

With this perspective article, we propose a three-tier model of community-based model of response to a humanitarian crisis to offload hospital systems and avoid their collapse.

We provide a pandemic model of care with pragmatic, understandable, and standardized protocols to manage many COVID-19 patients out of hospital. The main goal of this approach is to avoid overloading and cross-contamination of the healthcare facilities by SARS-CoV-2, with concomitant reduction of the ability to provide acute and routine care.

The main limitation of this perspective article is the absence of data supporting the validity of the model presented. Nevertheless, proactive and narrative engagement of health workers and policy makers from the frontline to empowers people and community and promote multisectoral policy and action for health, has been advocated as an essential foundation of PHC in emergency setting to improve outcome of the patients and the communities (World Health Organization, 2020c).

In the declination of some details and of the implementation process, this model is based on the territorial features of the province of Bergamo; a pilot experience should precede the full implementation of the model. Adjustments and amendments are required to fit different contexts. We hope that this thought effort will trigger discussions and guide strategic choices in other world regions, during new resurgences, and in future epidemics as well.

## Data Availability Statement

The original contributions presented in the study are included in the article/supplementary material. Further inquiries can be directed to the corresponding author.

## Author Contributions

All the authors contributed fairly to the writing of the paper. All authors contributed to the article and approved the submitted version.

## Conflict of Interest

The authors declare that the research was conducted in the absence of any commercial or financial relationships that could be construed as a potential conflict of interest.
